# The Infection Process of *Yersinia ruckeri*: Reviewing the Pieces of the Jigsaw Puzzle

**DOI:** 10.3389/fcimb.2018.00218

**Published:** 2018-06-26

**Authors:** José A. Guijarro, Ana I. García-Torrico, Desirée Cascales, Jessica Méndez

**Affiliations:** Área de Microbiología, Departamento de Biología Funcional, Facultad de Medicina, Instituto de Biotecnología de Asturias (IUBA), Universidad de Oviedo, Oviedo, Spain

**Keywords:** *Yersinia ruckeri*, infection route, virulence genes, comparative genome, proteome analysis

## Abstract

Finding the keys to understanding the infectious process of *Yersinia ruckeri* was not a priority for many years due to the prompt development of an effective biotype 1 vaccine which was used mainly in Europe and USA. However, the gradual emergence of outbreaks in vaccinated fish, which have been reported since 2003, has awakened interest in the mechanism of virulence in this pathogen. Thus, during the last two decades, a large number of studies have considerably enriched our knowledge of many aspects of the pathogen and its interaction with the host. By means of both conventional and a variety of novel strategies, such as cell GFP tagging, bioluminescence imaging and optical projection tomography, it has been possible to determine three putative *Y. ruckeri* infection routes, the main point of entry for the bacterium being the gill lamellae. Moreover, a wide range of potential virulence factors have been highlighted by specific gene mutagenesis strategies or genome-wide transposon/plasmid insertion-based screening approaches, such us *in vivo* expression technology (IVET) and signature tagged mutagenesis (STM). Finally, recent proteomic and whole genomic analyses have allowed many of the genes and systems that are potentially implicated in the organism's pathogenicity and its adaptation to the host environmental conditions to be elucidated. Altogether, these studies contribute to a better understanding of the infectious process of *Y. ruckeri* in fish, which is crucial for the development of more effective strategies for preventing or treating enteric redmouth disease (ERM).

## Introduction

*Yersinia ruckeri* is able to infect different fish species such as carp, catfish, sturgeon, perch and burbot (Tobback et al., 2007; Kumar et al., 2015), although it mainly affects salmonids. Interestingly, *Y. ruckeri* has recently been isolated as an unusual microorganism in a human wound infection (De Keukeleire et al., 2014) as well as in milk, cheese, chicken and minced meat (Özdemir and Arslan, 2015). In salmonids it causes enteric red mouth disease (ERM), which is important owing to the economic losses it causes in the aquaculture industry. ERM is a systemic disease affecting fish in all stages of development, causing a high degree of mortality. The disease occurs acutely in juveniles and has a tendency to become chronic in adult fish (Tobback et al., 2007). Some infected fish are asymptomatic, becoming carriers of the pathogen and acting as reservoirs of the bacterium. It is important to highlight that *Y. ruckeri* is a facultative intracellular bacterium, capable of surviving inside macrophages (Ryckaert et al., 2010).

*Y. ruckeri* strains have been classified into four serotypes (Romalde et al., 1993), the main outbreaks in fish farms being caused by serotype O1 biotype I, although other serotypes, particularly serotype O2, can also be involved (Romalde et al., 2003). Vaccination is the most effective way to control the disease. ERM biotype 1 vaccine, which was the first to be available for fish (Amend et al., 1983), has been used since the early 80s and is very effective in the prevention of the disease. However, during recent years worldwide outbreaks have been produced mainly by novel non-motile and lipase-negative strains, belonging to biotype II (Austin et al., 2003; Fouz et al., 2006; Arias et al., 2007; Calvez et al., 2014). These phenotypes have no appreciable effect on the virulence of the pathogen (Evenhuis et al., 2009; Welch et al., 2011), but the biotype 2 strains are able to elude the protection provided by the vaccine, probably due to the antigenic differences existing in their O-antigen with respect to biotype 1 strains (Tinsley et al., 2011). This has promoted the development and subsequent commercialization of a new vaccine which simultaneously confers protection against the two biotypes (Tinsley et al., 2011).

Owing to the prompt development of the vaccine, the study of the virulence mechanisms of this bacterium has been side-lined for a long time. Nevertheless, during the last few years a considerable number of reports related to the route of *Y. ruckeri* infection or its virulence, as well as several comparative genomic and proteomic analyses have been published. All of this knowledge about the infectious process of this bacterium is condensed and critically reviewed in the present article.

## *Yersinia ruckeri* infection route

Studies aimed at determining the infection path and invasion mechanism of fish by *Y. ruckeri* have been carried out with different strains, modes of infection and experimental conditions, which sometimes makes it difficult to draw a conclusion. In addition, various tools have been developed and used for elucidating the interaction between *Y. ruckeri* and rainbow trout, its natural host.

**Figure 1:**
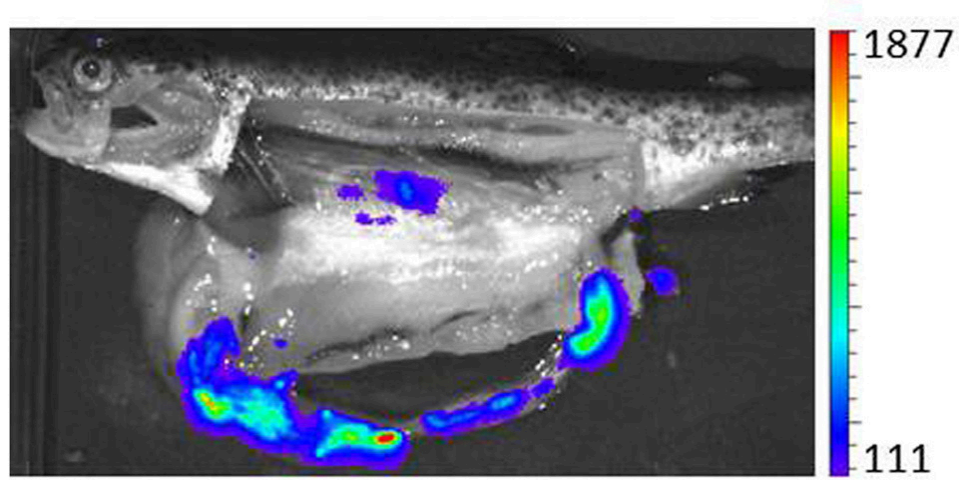
Bioluminescent tracking of *Y. ruckeri* harboring pCS26-Pac in a fish infected by bath immersion with 10^7^ cfu ml^−1^ for 1 h. Bioluminescence emitted by the bacterium was captured three days postinfection by Ivis-Lumina equipment. Color standards represent “RLU max.” This measure expresses the highest number of counts in a pixel inside the region analyzed (Figure taken from Méndez and Guijarro, 2013).

Bacterial counting in fish tissues at different times after experimental infection (Tobback et al., 2009, 2010); GFP tagging of *Y. ruckeri* and further visualization by epifluorescence or flow cytometry in tissues (Welch and Wiens, 2005); *in situ* hybridization (Khimmakthong et al., 2013); bioluminescence imaging using strains harboring the *luxCDABE* operon from *Photorhabdus luminescens* (Méndez and Guijarro, 2013) and optical projection tomography (OPT) (Ohtani et al., 2014), a recently developed three-dimensional bioimaging technique combined with immunohistochemical assay (IHC) (Tobback et al., 2009; Ohtani et al., 2014), have all been used. Each of these technologies provides a different approach to determining the route of entry and tissue distribution of *Y. ruckeri*. Although all of them have limitations, if we analyse the results obtained as a whole, it seems that the gills are the portal of entry for the bacterium into the fish. In effect, most studies have established this organ as the first to be infected (Tobback et al., 2009; Khimmakthong et al., 2013; Ohtani et al., 2014, 2015), and more specifically the pavement cells of the gill lamellae, from which the bacterium would spread to blood (Ohtani et al., 2014). Thus, *Y. ruckeri* was detected only 1 min post infection in the blood, where the number of bacteria increased for 40 min, reaching 2.3x10^5^ CFU/ml (Ohtani et al., 2014). Although gills seem to be the main point of entry of *Y. ruckeri*, other sites in the fish cannot be excluded. In effect, most studies establish the presence of *Y. ruckeri* in the intestine shortly after infection (Khimmakthong et al., 2013; Ohtani et al., 2014, 2015). It is known that the mucus of the gills is twice as efficient as that of the intestine in enabling the adherence of the bacterium (Tobback et al., 2010). Moreover, after infection, twice as many bacteria were detected in the gills as in the intestine (Tobback et al., 2009; Khimmakthong et al., 2013). However, bacteria have been detected in the intestine from 1 min (Khimmakthong et al., 2013) to 30 min post-infection (Ohtani et al., 2014). Additionally, intestinal dissemination is the main sign of the pathology (Figure 1) (Méndez and Guijarro, 2013; Ohtani et al., 2014). This, together with the fact that it is unusual for an intestinal pathogen to enter the host by any other route than the oral one means that the digestive tract cannot be excluded as another portal of entry. In addition, Ohtani et al. (2014) used OPT analysis to establish a third route of infection for *Y. ruckeri*, through the skin surface on the lateral line, from which it would penetrate to internal cell layers, a similar result to that found by Khimmakthong et al. (2013). A recent study reported the ability of two isolates of *Y. ruckeri* to invade different types of salmon epithelial cells (Menanteau-Ledouble et al., 2018), the biotype 1 isolate (ATCC 29473) being more infectious than the non-motile biotype 2 strain (A7959-11). The authors suggested that the bacterium could enter host cells by means of several mechanisms that take advantage of cytoskeletal systems. The capacity to invade the epithelium would undoubtedly facilitate entry into the host.

In conclusion, all the results indicate that *Y. ruckeri* can access the interior of rainbow trout by at least three different paths: the gills, lateral line and digestive tract. From these locations bacteria would spread to the blood circulation system to further colonize and infect other internal organs, causing in many cases the death of the fish.

## Defined genes related to virulence

**Figure 2:**
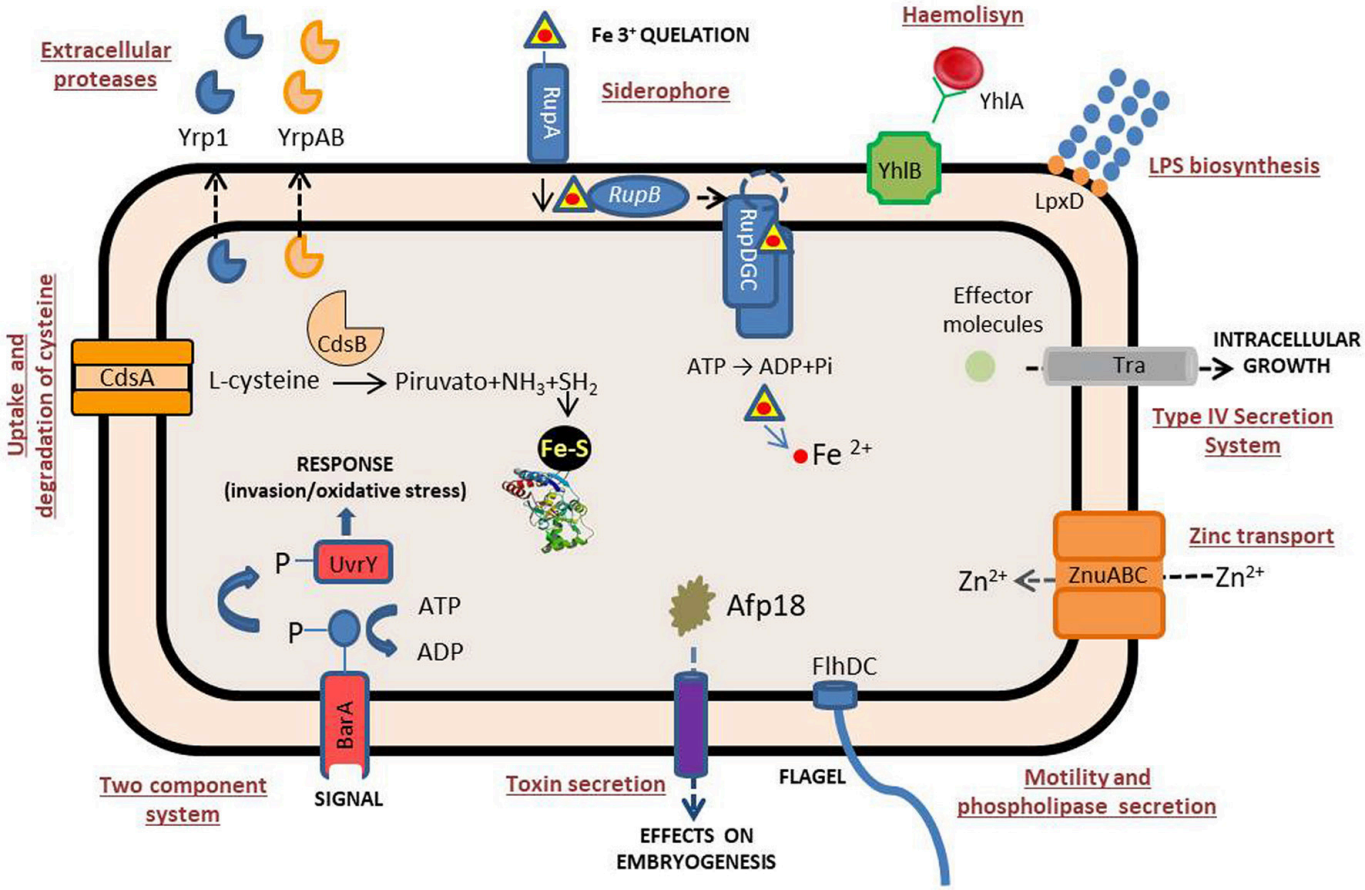
Schematic representation of the *Y. ruckeri* systems related to virulence. See text for further details.

Despite its being a microorganism responsible for a high proportion of losses in continental aquaculture, there are few studies on *Y. ruckeri* virulence factors, perhaps as a consequence of the early development of a vaccine, which made their study unattractive. Many of the virulence factors described so far refer to extracellular factors (ECF) that are common to a wide range of gram-negative pathogenic bacteria, particularly the enterobacteria group (Table 1, Figure 2). Indeed, extracellular proteases, haemolysins and siderophores are among the main extracellular molecules involved in the *Y. ruckeri* infection process.

**Table 1:** Virulence related genes of *Y. ruckeri*.

**Gene**	**Function**	**References**
*yrp1*	Extracellular protease	Secades and Guijarro, 1999
*yrpAB*	Peptidases	Navais et al., 2014b
*rucC*	Iron captation. Ruckerbactin Siderophore synthesis	Fernandez et al., 2004
*yhlA*	Hemolysine	Fernández et al., 2007b
*traI*	Type IV secretion system	Méndez et al., 2009
*cdsBA*	Cysteine transport and degradation	Méndez et al., 2011
*znuA*	Zinc transport	Dahiya and Stevenson, 2010b
*uvrY*	Response regulator. Two component system	Dahiya and Stevenson, 2010c
*lpxD*	Lipid A biosynthesis	Altinok et al., 2016
*afp18*	Toxin. Glycosyltransferase	Jank et al., 2014
*flhD*	Motility regulation	Jozwick et al., 2016

Yrp1 is a serralysin family extracellular metalloprotease, widely characterized from an enzymatic point of view (Secades and Guijarro, 1999) and secreted through an ABC transport system (Fernández et al., 2002). It is able to degrade different matrix and muscle proteins such as laminin, fibrinogen, gelatin, actin, and myosin (Fernandez et al., 2003). Although it was shown not to be present in all virulent *Y. ruckeri* strains (Secades and Guijarro, 1999), its presence makes the bacteria more virulent. Transcriptional fusion between the *luxCDABE* operon and the *yrp1* promoter demonstrated that *yrp1* is induced more *in vivo* than *in vitro* (Méndez and Guijarro, 2013). This is consistent with the expression of *yrp1* in fish tissues and the observation that an *yrp1* mutant strain is significantly attenuated in virulence (Fernández et al., 2002). Interestingly, a heat-inactivated Yrp1 toxoid was able to elicit a strong protection against the ERM disease (Fernandez et al., 2003). All of this established a clear and important role for this protease in the pathogenic process.

Additional degrading enzymes were identified in *Y. ruckeri*, such as *two* putative peptidases belonging to the U32 family which are encoded by the *yrpAB* operon (Navais et al., 2014b). Both genes, *yrpA* and *yrpB*, are present in a similar genetic organization in different pathogens of the enterobacteria group, including human pathogenic *Yersinia* species (Navais et al., 2014b). A synergistic effect occurred in the induction of the *yrpAB* operon under low oxygen conditions when peptones were also present in the culture medium (Navais et al., 2014b). Infection studies with a *yrpA* mutant strain suggest that the YrpA peptidase contributes to virulence in a similar way to the U32 peptidases of *Proteus mirabilis* (Zhao et al., 1999) and *Helicobacter pylori* (Kavermann et al., 2003). A more detailed analysis of the role of these peptidases in the virulence of pathogenic *Yersiniae* could be interesting from a public health point of view.

Two different genetic approaches have been applied to allow the selection and further identification of *Y. ruckeri* genes related to the infection process: *in vivo* expression technology (IVET) (Fernandez et al., 2004) and signature-tagged mutagenesis (STM) (Dahiya and Stevenson, 2010a). Each of these has advantages and disadvantages, but undoubtedly, their application has led to the identification of a number of genes related to *Y. ruckeri* pathogenesis.

IVET allowed the identification of up to 14 genes specifically induced *in vivo*, during the infection process of *Y. ruckeri*. Among them, those related to two-component and type IV secretion systems, adherence, haemolytic activity and iron acquisition were further analyzed. It has been found that the genes involved in iron acquisition through the catechol siderophore ruckerbactin have a genetic organization similar to those of the *E. coli* enterobactin gene cluster (Fernandez et al., 2004). Regulation of this cluster depends on iron availability and temperature in such a way that low iron conditions and low temperature (18°C in relation to 28°C) induced its expression (Fernandez et al., 2004). It was shown that production of the siderophore is upregulated during the infection of fish (Fernandez et al., 2004; Fernández et al., 2007a) and it is also involved in virulence since 100-fold attenuation, in relation to the parental strain, was obtained when an isogenic mutant in *rucC*, a gene from the ruckerbactin cluster, was used in LD_50_ experiments (Fernandez et al., 2004).

A putative additional system of *Y. ruckeri* for obtaining iron is the *yhlBA* cluster, also identified by IVET. The *yhlA* gene encodes a *Serratia*-type haemolysin, whereas the *yhlB* product is related to the secretion/activation of YhlA (Fernández et al., 2007b) (Figure 2). Analysis of *yhlB::lacZY* transcriptional fusion indicated that higher levels of expression of *yhlB* were obtained under iron-starvation conditions, and also at 18°C than at 28°C (Fernández et al., 2007b). Interestingly, the involvement of the two genes in bacterial virulence is indicated not only by the virulence attenuation obtained when *yhlA* and *yhlB* insertional mutants were used, but also by the cytolytic properties of YhlA against the BF-2 fish cell line (Fernández et al., 2007b). *In vivo* monitoring of *yhlBA* promoter showed that it was highly expressed (Méndez and Guijarro, 2013), as was expected, since the system had been previously isolated as an IVET clone. In contrast to *yrp1*, the *yhlAB* operon is present and the haemolytic activity is detectable in different *Y. ruckeri* strains from different origins and geographical locations. This indicates that this operon is of importance for virulence in this species (Fernández et al., 2007b).

The analysis of a chromosomally located *traHIJKCLMN* operon of *Y. ruckeri* that was identified by IVET showed that it was structurally related to the DNA transfer system present in the pADAP virulence-related plasmid of *Serratia entomophila* (Hurst et al., 2003). The cluster was present in different *Y. ruckeri* strains but it was not found in the genomic analysis of human pathogenic *Yersinia* (Méndez et al., 2009). This operon is composed of at least eight genes displaying homology with type IV secretion systems (T4SS). Indeed, *traH, traI, traJ*, and *traK* genes encoded for proteins similar to those encoded by the *dot/icm* genes of the T4SS of *Legionella pneumophila* (Komano et al., 2000). Thus, it is likely that the gene products of the *tra* operon constitute parts of the T4SS involved in the transfer of virulence factors. In fact, insertional mutagenesis of the *traI* gene resulted in attenuation of the virulence (Méndez et al., 2009). Like other virulence factors, the *tra* operon was more highly expressed at 18°C than at 28°C.

A novel two-gene operon *cdsBA* involved in the transport and further degradation of L-cysteine was also selected by IVET. Attenuation of a *cdsA* mutant supported a role in virulence (Méndez et al., 2011). The *cdsB* gene encoded an L-cysteine desulfidase involved in cysteine degradation, previously characterized in the archaeal *Methanocaldococcus jannaschii* (Tchong et al., 2005), whereas *cdsA* is a cysteine permease (Méndez et al., 2011). Interestingly, the operon was shown to be present in several anaerobic and facultative bacterial groups, particularly in some species of enterobacteria, including *Y. enterocolitica*, although it was absent in *Y. pseudotuberculosis* and *Y. pestis* (Méndez et al., 2011). This operon was specifically induced by L-cysteine under low oxygen conditions and the uptake of this amino acid was an energy-dependent process (Méndez et al., 2011). Transcriptional fusion of the *cdsBA* promoter with the *luxCDABE* operon showed that the *cdsA* and *cdsB* genes were barely expressed under routine laboratory conditions but were found to have high activity inside the fish. Therefore, virulence attenuation of the *cdsA* mutant underlines the importance of this operon for the bacterium during the infection process. Although the precise role of the CdsAB system remains unknown, it was suggested that it could be involved in the generation of iron-sulfur-centers for FE-S proteins or in the accumulation of glutathione, an important detoxification and redox buffer molecule in the cell (Méndez et al., 2011).

Interestingly, more of these IVET-selected genes are induced at 18°C, the temperature around which outbreaks of the disease take place, than at 28°C, the optimal temperature for growth of the bacterium (Fernández et al., 2007b; Méndez et al., 2009). This finding points to the existence in *Y. ruckeri* of specific mechanisms (thermo-induced changes of DNA supercoiling, RNA and protein thermometer, etc.) for the temperature regulation of virulence at temperatures below the optimal for its growth, as seems to occur in a good number of microorganisms that infect ectothermic animals (Guijarro et al., 2015).

STM was also able to select *Y. ruckeri* mutants that can survive *in vitro* but not in the fish host (Dahiya and Stevenson, 2010a). Further identification of the inactivated genes in these mutants revealed up to 25 different ORFs (Dahiya and Stevenson, 2010a), among which the *znuA* gene, coding a zinc binding protein, and the *uvrY* gene, coding a response regulator, were chosen for deeper study. The *znuA* gene forms part of the *znuABC* operon encoding a high-affinity zinc transporter, which has a genetic organization similar to that found in *E. coli* (Patzer and Hantke, 2000) and *S*. Typhimurium (Campoy et al., 2002). In fact, the introduction of the *Y. ruckeri znuABC* locus was able to restore the growth of a *znuABC* mutant of *E. coli* in zinc-deficient media (Dahiya and Stevenson, 2010b). The *znuABC* system is widely distributed in bacteria such as *Brucella abortus* (Kim et al., 2004), *Campylobacter jejuni* (Davis et al., 2009) and *Neisseria gonorrhoeae* (Lim et al., 2008), contributing to their virulence. Interestingly, mutations in the *znuABC* system of *Y. pestis* did not affect virulence (Bobrov et al., 2014, 2017). The reason is that this bacterium has an additional zinc transport system mediated by some components of the yersiniabactin siderophore (Ybt) which is able to sequestrate zinc in addition to iron (Bobrov et al., 2014, 2017). In mouse models of bubonic and pneumonic plague this system compensates for the loss of *znuABC*. However, the simultaneous absence of both systems leads to a decrease in *Y. pestis* virulence (Bobrov et al., 2014, 2017). However, in *Y. ruckeri* it seems that the *znuABC* system is the main Zn acquisition system, since the *znuA* mutant had nearly 150- to 350-fold lower infection loads in the kidney of rainbow trout than the parental strain, which demonstrates that this Zn transport system has an important role in the infection process (Dahiya and Stevenson, 2010b).

In another mutant selected by STM, the BarA-UvrY two-component system was inactivated. This mutant was recovered from fish in 5- to 15-fold lower numbers compared to the parental strain at the beginning of the infection (Dahiya and Stevenson, 2010c), suggesting that this system is involved in the invasion of gills or gut tissues, as occurred in the *uvrY* mutants of *Y. pseudotuberculosis* (Heroven et al., 2008), *E. coli* (Herren et al., 2006) and *S*. Typhimurium (Johnston et al., 1996). In addition, the *Y. ruckeri uvrY* mutant was more sensitive to H_2_O_2_, which suggests an increasing susceptibility to oxidative killing by phagocytic cells. All of this resulted in a lower recovery (10^3^- to 10^5^-fold) of the *uvrY* mutant than the parental strain at the end of co-infection experiments which were carried out by immersion of rainbow trout in water containing both strains (Dahiya and Stevenson, 2010c).

As expected, LPS is important for the pathogenesis of *Y. ruckeri* (Altinok et al., 2016). Specific deletion of the *lpxD* gene, involved in the lipid A biosynthesis, resulted in an LPS-deficient strain. This *lpxD* mutant showed a significant attenuation of virulence (10^3^-fold) in relation to the parental strain when LD_50_ determination was carried out by injection (Altinok et al., 2016). It is important to highlight that *lpxD* mutation reduced the overall number of invasive bacteria, which in turn, may influence the process of invasion and colonization, leading to a decrease in virulence. Special attention must be paid to a virulence factor related to legionaminic acid, a component of the *Y. ruckeri* LPS that will be addressed in the next section.

Another interesting virulence factor that has been described in *Y. ruckeri* is the Afp18 toxin, although it should be noted that its effect has hitherto only been tested in zebrafish embryos (Jank et al., 2014). This is encoded by a gene that is part of the antifeeding prophage gene cluster (*afp*), consisting in a 25 Kb DNA segment involved in the production of a prophage contractile tail. This cluster is similar to the one found in *Serratia entomophila* (Rybakova et al., 2013; Jank et al., 2014), which has common characteristics with the R-pyocins type VI secretion system delivery apparatus of *Pseudomonas aeruginosa* (Heymann et al., 2013). This contractile tail is thought to capture the Afp18 toxin and inject it into the cell cytoplasm of zebrafish blastoderm cells (Jank et al., 2014). The Afp18 protein has a glycosyltransferase domain. Its activity alters the early phase of zebrafish embryogenesis, whereas a non-functional Afp18 lacking the glycosyltransferase domain has no effect on development (Jank et al., 2014). The effect of the Afp18 glycosyltransferase is through a process of specific tyrosine mono-O-GlcNAcylation of RhoA, a regulatory GTPase associated with cytoskeleton development, resulting in actin depolymerization and the abrogation of early zebrafish development (Jank et al., 2014). Interestingly, genome analysis indicates that this antifeeding prophage system is widely distributed among prokaryotes and archaea (Sarris et al., 2014). In *S. entomophila* the Afp18 toxin acts as a virulence factor during the infection of insects (Rybakova et al., 2013), and could also be important as a virulence factor in human pathogenic bacteria. Since all this work was carried out on zebrafish embryos, it could be interesting to study the role of Afp18 on *Y. ruckeri* infection of rainbow trout by generating a mutant strain and following the progression of the disease.

Finally, two elements that should be taken into account are the *flhDC* operon, whose inactivation results in a more virulent strain and the heat sensitive factor (HSF), which has been considered as a virulence factor for a good number of years. The *flhDC* is a flagellar master operon of *Y. ruckeri* involved in the regulation of motility and phospholipase secretion, among other processes (Jozwick et al., 2016). In fact, mutation of the *flhD* gene resulted in significant changes in the bacterial transcriptome (Jozwick et al., 2016). Interestingly, no differences were found between the *flhD* mutant and the wild type strain when a standard experimental challenge model with mortalities as an endpoint was used. However, there was a significant increase in the *flhD* mutant density within the spleen in relation to the wild type strain in competition experiments, a characteristic which was reverted in the complemented strain (Jozwick et al., 2016). The result indicated that mutation of the *flhDC* operon gives a competitive advantage over the parental strain during the infection process. Although it is not known how the mutation in this operon affects bacterial virulence, the result is similar to that found in the *S*. Typhimurium *flhD* mutant in the sense that it was more virulent than the parental strain (Schmitt et al., 2001).

It is also worth mentioning that for many years it was thought HSF was required for virulence in *Y. ruckeri* (Furones et al., 1993). However, this assumption was recently discarded, since HSF was found to be an alkylsulphatase enzyme encoded by the *yraS* gene, whose mutation did not produce any modification in the virulence of the strain in relation to the wild type (Navais et al., 2014a).

## Predicted virulence factors deduced from proteomic and genomic analysis

During recent years the development of alternative technologies based on proteomic, transcriptomic and genomic analyses has provided new tools to identify potential virulence factors in bacteria. It is important to consider that this type of analysis is based on comparison and only predicts the potential role of some of the genes or proteins identified as virulence factors. In *Y. ruckeri*, proteomic studies have been carried out under very specific culture conditions. Thus, differentially regulated proteins were identified and quantified in normal and iron-limited media using a label-free, gel-free shotgun proteomic approach (Kumar et al., 2016). Iron is an essential nutrient for bacteria that is difficult to obtain during the infection process, so a limited-iron environment is expected to induce a set of genes, some of which are related to pathogenesis. Under reduced iron availability, the majority of up-regulated proteins in biotype 1 and biotype 2 *Y. ruckeri* strains were related to iron-binding proteins (YfuA, YiuA and YfeA), iron-binding transporters (HemS and Feo), hemin transport (YhlBA) and TonB-dependent iron binding receptor, the latter probably being involved in the transport of ruckerbactin into the periplasm of cells and siderophore biosynthesis proteins. YfeA and YhlBA systems have previously been described as important virulence factors in *Y. pestis* (Bearden and Perry, 1999) and *Y. ruckeri* (Fernández et al., 2007b), respectively. Another up-regulated protein under iron-limited conditions was a superoxide dismutase, involved in protective mechanisms against oxidative stress, and particularly important in intracellular pathogens such as *Y. ruckeri*. Interestingly, this study revealed no significant differences in protein profile, using liquid chromatography-mass spectrometry analyses, between motile and non-motile *Y. ruckeri* strains, corresponding with biotype 1 and biotype 2, when they were cultured under iron-limited conditions, indicating that iron availability it is not involved in the differential virulence behavior of the two biotypes (Kumar et al., 2016).

A similar study was conducted in order to establish the differential protein profile between virulent and avirulent strains of *Y. ruckeri* grown under standard conditions (Kumar et al., 2017). Interestingly, in virulent strains, a total of 16 proteins were shown to be upregulated, including the haemolysis expression regulator HtrA, DegQ proteases, an anti-sigma regulator factor, different transcriptional regulators belonging to the LuxR, AsnC, and PhoP families, RNA-binding protein Hfq, invasion protein Inv, Cu-Zn superoxide dismutase, among others. All of these have been previously described as virulence-related proteins in different bacterial pathogens, which strongly suggests a role for them in the infection process of *Y. ruckeri*.

Invasins play an essential role in the initial colonization and attachment of *Y. enterocolitica* and *Y. pseudotuberculosis* (Chauhan et al., 2016). In *Y. ruckeri*, invasin (*yrInv*) and invasin-like molecule (*yrllm*) are two putative inverse autotransporter genes that, according to qRT-PCR analysis, were upregulated in a medium that mimics the environmental conditions present in the fish host (Wrobel et al., 2018), so they are likely to be involved in the infection process of *Y. ruckeri* although there are no conclusive data.

Whole genome analysis of *Y. ruckeri* strains provides a new approach to identifying putative virulence factors. Indeed, genome analysis of the *Y. ruckeri* SC09 strain isolated from catfish (*Ictalurus punctatus*) showed that it harbors a *ysa* locus containing all the genetic components of a type III secretion system (T3SS) quite similar in gene sequence and genetic structure to the *Salmonella enterica* pathogenicity island 1 and chromosomally encoded T3SS of *Y. enterocolitica* 1B (Liu et al., 2016). It should be remembered that T3SS are present only in virulent strains of human pathogenic *Yersinia* (Chen et al., 2010). For this reason, it is possible that the T3SS of *Y. ruckeri* contributes to the survival of the bacterium in fish macrophages and helps in the invasion of gill and gut epithelia. Other putative systems in *Y. ruckeri* SC09 that, according to the genomic analysis carried out by Liu et al. (2016), could be involved in the development of the infection in fish are: a type II secretion system, different two-component signal transduction systems and a phage shock protein system (Psp), which is distributed among enterobacteria and is implicated in the virulence of *Salmonella, Shigella* and *Yersinia* species (Huvet et al., 2011). Interestingly, a type IV secretion system described as a putative virulence factor by Liu et al. (2016) is unique to *Y. ruckeri* SC09 strain and it is not present in the other four *Y. ruckeri* strains analyzed by Cascales et al. (2017).

The comparative analysis of the genome sequences of five *Y. ruckeri* strains showed that, although they share approximately 75% of their genes, significant genetic differences were detected depending on the serotype or virulence of these strains (Cascales et al., 2017). Thus, a cluster of 18 genes related to the biosynthesis of legionaminic acid, the major component of the LPS, was exclusively present in serotype O1 strains. This cluster was absent in serotype O2 strains as well as in other *Yersinia* species, but present in different aquatic pathogenic bacteria such as *Vibrio vulnificus, Aeromonas salmonicida, Vibrio fischeri* (Cascales et al., 2017) and also in *Campylobacter jejuni*, where it is involved in virulence (Zebian et al., 2016). In addition to this cluster, serotype O1 strains share a total of 268 genes, among which are the invasin and the type IV secretion system corresponding with the *traHIJKCLMN* operon (Méndez et al., 2009).

These genes shared by serotype O1 strains could adapt the bacterium to a particular host, since the three serotype O1 strains analyzed (150, CSF007-82, and ATCC29473) were isolated from rainbow trout. In the same way, a serotype O2 strain (Big Creek 74) together with *Y. ruckeri* SC09, a putative O2 strain, which were isolated from chinook salmon and catfish respectively, share 122 genes which are absent in serotype O1 strains. These include a cluster involved in fimbriae biosynthesis similar to the *Stf* cluster of *S*. Typhimurium, associated with virulence (Emmerth et al., 1999) and an insecticidal toxin complex similar to the one found in *Vibrio parahaemolyticus*, involved in hepatopancreatic necrosis disease in penaeid shrimps (Tang and Lightner, 2014).

**Figure 3:**
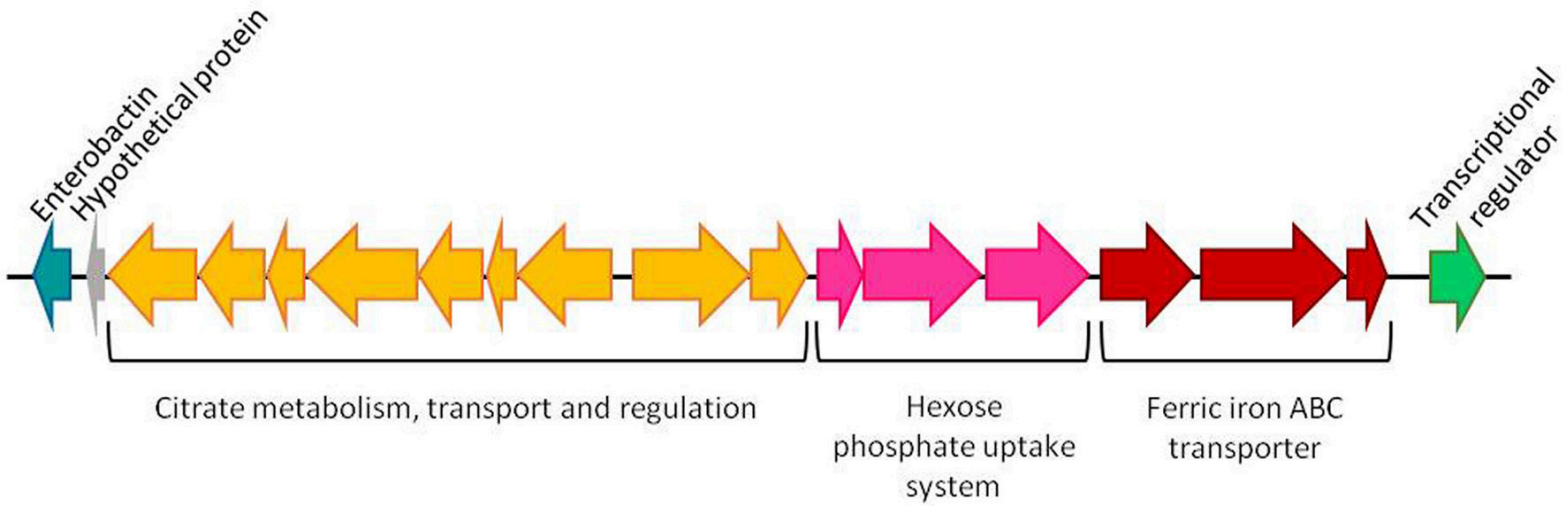
Cluster of genes absent from *Y. ruckeri* ATCC29473 type strain and present in the strains *Y. ruckeri* 150, CSF007-82, Big Creek and SC09. The region contains genes encoding for an enterobactin-like siderophore (blue), nine genes involved in the uptake and metabolism of citrate (yellow), a group of three genes related to hexose phosphate uptake (pink), three genes involved in iron transport (red) and a Crp-Fnr family transcriptional regulator (green). (1) Citrate succinate antiporter, (2) 2-(5′-triphosphoribosyl)-3′-dephosphocoenzyme-A synthase, (3) Apo-citrate lyase phosphoribosyldephospho-CoA transferase, (4) Citrate lyase alpha chain, (5) Citrate lyase beta chain, (6) Citrate lyase gamma chain acyl carrier protein, (7) [Citrate[pro-3S]-lyase] ligase, (8) Sensor kinase, (9) Transcriptional regulatory protein, (10) Transcriptional regulatory protein, (11) Sensor histidine protein kinase glucose-6-phosphate specific, (12) Hexose phosphate uptake regulatory protein, (13) Ferric iron ABC transporter iron-binding protein, (14) Ferric iron ABC transporter permease protein, (15) Ferric iron ABC transporter binding subunit (Figure taken from Cascales et al., 2017).

In the study of Cascales et al. (2017) it is proposed that the characteristic attenuation in virulence of the ATCC29473 type strain is a consequence of the lack of 21 genes that were present in the other virulent strains. It should be noted that 17 out of these 21 genes are clustered in all four genomes analyzed (Figure 3). This region includes genes encoding a Crp-Fnr family transcriptional regulator, enzymes related to enterobactin-like siderophore and gene clusters involved in iron transport, hexose phosphate uptake and citrate metabolism. Most of these genes are related to virulence in different bacteria (Gray et al., 2006; Urbany and Neuhaus, 2008; Moisi et al., 2013) so the absence of this region could be responsible for the attenuation of the type strain.

## Conclusions and further studies

The results lead to the conclusion that there exist at least three different routes of infection by *Y. ruckeri*. The most important pathway for the development of ERM has still to be elucidated, but it seems clear that, although the primary bacteremia is generated by the entry of the bacterium into the fish through the gills, the very significant presence of the bacterium in the intestine and also the signs of the disease, strongly suggest that the digestive route must play a relevant role. The virulence of *Y. ruckeri* is multifactorial and it depends not only on the presence of certain genes in each strain but also on certain environmental factors, particularly, temperature. In fact, a temperature below the optimal for bacterial growth and close to the one at which outbreaks occur seems to be important in the expression of some of the genes encoding virulence factors. This appears to be one of the areas for future research, since presently unknown regulation systems could be involved in this temperature-dependent response. Although a lot of genes involved in the pathogenesis of the bacterium have recently been identified, there are still many points that need to be addressed, such us the temporal and spatial expression pattern of each virulence factor and their specific function or the identification of the regulatory mechanisms which modulate the virulence gene expression. The new techniques and methodologies developed in recent years, including CLIQ-BID, a method allowing quantification of damage in eukaryotic cells infected by bacteria (Wallez et al., 2018), the new RNA-seq techniques such as Term-seq (Dar et al., 2016) and Dual RNA-seq (Westermann et al., 2016), useful for the analysis of pathogens under complex environmental conditions and Single Cell RNA-seq (Avraham et al., 2015; Saliba et al., 2017) can help to develop different approaches. Moreover, analysis of the data provided by the genetic and proteomic studies may be a good starting point for elucidating the role of new genes in the infectious process of *Y. ruckeri*. In fact, two specific gene clusters should be considered for their potential role in virulence. This is the case of a cluster involved in legionaminic acid biosynthesis that could be important in the pathogenesis of serotype O1 strains, and the 21 genes absent from the virulence-attenuated *Y. ruckeri* type strain, some of them encoding putative virulence factors.

## Author contributions

JG and JM drafted the first version of the manuscript. DC, JM, AG-T, JG contributed to bibliography analysis and manuscript improvement. All authors have reviewed the final version of the manuscript.

### Conflict of interest statement

The authors declare that the research was conducted in the absence of any commercial or financial relationships that could be construed as a potential conflict of interest.
